# Supplementary material for: HIVprotI: an integrated web based platform for prediction and design of HIV proteins inhibitors
Source: J Cheminform. 2018 Mar 9;10:12. doi: 10.1186/s13321-018-0266-y (PMC5845081; doi:10.1186/s13321-018-0266-y)
Supplement: Supplementary file 2 — Additional file 2. Source code of HIVProtI web server. [file 13321_2018_266_MOESM2_ESM.zip › HIVprotI_Source-code/hivprotI/weka-3-6-6/doc/weka/core/Debug.DBO.html]

Debug.DBO


---


|  |  |  |
| --- | --- | --- |
| **Overview** \| **Package** \| **Class** \| **Tree** \| **Deprecated** \| **Index** \| **Help** \| **Weka's home** | |  |
| **PREV CLASS**   **NEXT CLASS** | **FRAMES**    **NO FRAMES**     **All Classes** |  |
| SUMMARY: NESTED \| FIELD \| CONSTR \| METHOD | DETAIL: FIELD \| CONSTR \| METHOD |  |


---


## weka.core Class Debug.DBO

```
java.lang.Object
  weka.core.Debug.DBO
```

**All Implemented Interfaces:**: java.io.Serializable, RevisionHandler

**Enclosing class:**: Debug

---

``` public static class Debug.DBO extends java.lang.Object implements java.io.Serializable, RevisionHandler ```

contains debug methods

**Version:**
:   $Revision: 7519 $

**Author:**
:   Gabi Schmidberger (gabi at cs dot waikato dot ac dot nz)

**See Also:**: Serialized Form

---

| **Field Summary** | |
| --- | --- |
| `Range` | `m_outputTypes`             range of outputtyp |
| `boolean` | `m_verboseOn`             enables/disables output of debug information |


**Constructor Summary**

`Debug.DBO()`


| **Method Summary** | |
| --- | --- |
| `boolean` | `dl(int num)`             Return true if the debug level is set same method as outpuTypeSet but better name |
| `void` | `dp(int debugType, java.lang.String text)`             prints out text but only if debug level is set. |
| `void` | `dp(java.lang.String text)`             prints out text if verbose is on. |
| `void` | `dpln(int debugType, java.lang.String text)`             prints out text + endofline but only if parameter debug type is set. |
| `void` | `dpln(java.lang.String text)`             prints out text + endofline if verbose is on. |
| `java.lang.String` | `getOutputTypes()`             Gets the current output type selection |
| `java.lang.String` | `getRevision()`             Returns the revision string. |
| `void` | `initializeRanges(int upper)`             Initialize ranges, upper limit must be set |
| `boolean` | `outputTypeSet(int num)`             Return true if the outputtype is set |
| `static void` | `p(java.lang.String text)`             prints out text. |
| `static void` | `pln(java.lang.String text)`             prints out text + endofline. |
| `void` | `setOutputTypes(java.lang.String list)`             Switches the outputs on that are requested from the option O |
| `void` | `setVerboseOn()`             Set the verbose on flag on |

**Methods inherited from class java.lang.Object**

`equals, getClass, hashCode, notify, notifyAll, toString, wait, wait, wait`

**Field Detail**

### m\_verboseOn

```
public boolean m_verboseOn
```

:   enables/disables output of debug information

---


### m\_outputTypes

```
public Range m_outputTypes
```

:   range of outputtyp


**Constructor Detail**

### Debug.DBO

```
public Debug.DBO()
```


**Method Detail**

### setVerboseOn

```
public void setVerboseOn()
```

:   Set the verbose on flag on

---


### initializeRanges

```
public void initializeRanges(int upper)
```

:   Initialize ranges, upper limit must be set

    :   **Parameters:**: `upper` - upper limit

---


### outputTypeSet

```
public boolean outputTypeSet(int num)
```

:   Return true if the outputtype is set

    :   **Parameters:**: `num` - value that is reserved for a specific outputtype **Returns:**: return true if the output type is set

---


### dl

```
public boolean dl(int num)
```

:   Return true if the debug level is set
    same method as outpuTypeSet but better name

    :   **Parameters:**: `num` - value that is reserved for a specific outputtype **Returns:**: return true if the debug level is set

---


### setOutputTypes

```
public void setOutputTypes(java.lang.String list)
```

:   Switches the outputs on that are requested from the option O

    :   **Parameters:**: `list` - list of integers, all are used for an output type

---


### getOutputTypes

```
public java.lang.String getOutputTypes()
```

:   Gets the current output type selection

    :   **Returns:**: a string containing a comma separated list of ranges

---


### dpln

```
public void dpln(java.lang.String text)
```

:   prints out text + endofline if verbose is on.
    helps to make debug output commands more visible in text

    :   **Parameters:**: `text` - the text to print

---


### dpln

```
public void dpln(int debugType,
                 java.lang.String text)
```

:   prints out text + endofline but only if parameter debug type is set.
    helps to make debug output commands more visible in text

    :   **Parameters:**: `debugType` - the type of the output: `text` - the text to print

---


### dp

```
public void dp(java.lang.String text)
```

:   prints out text if verbose is on.
    helps to make debug output commands more visible in text

    :   **Parameters:**: `text` - the text to print

---


### dp

```
public void dp(int debugType,
               java.lang.String text)
```

:   prints out text but only if debug level is set.
    helps to make debug output commands more visible in text

    :   **Parameters:**: `debugType` - the type of the output: `text` - the text to print

---


### pln

```
public static void pln(java.lang.String text)
```

:   prints out text + endofline.
    helps to make debug output commands more visible in text

    :   **Parameters:**: `text` - the text to print

---


### p

```
public static void p(java.lang.String text)
```

:   prints out text.
    helps to make debug output commands more visible in text

    :   **Parameters:**: `text` - the text to print

---


### getRevision

```
public java.lang.String getRevision()
```

:   Returns the revision string.

    :   **Specified by:**: `getRevision` in interface `RevisionHandler`
    :   **Returns:**: the revision


---


|  |  |  |
| --- | --- | --- |
| **Overview** \| **Package** \| **Class** \| **Tree** \| **Deprecated** \| **Index** \| **Help** \| **Weka's home** | |  |
| **PREV CLASS**   **NEXT CLASS** | **FRAMES**    **NO FRAMES**     **All Classes** |  |
| SUMMARY: NESTED \| FIELD \| CONSTR \| METHOD | DETAIL: FIELD \| CONSTR \| METHOD |  |


---
